# Annotations

**Published:** 1897-07-10

**Authors:** 


### ANNOTATIONS.

### The Vital Statistics of Block Dwellings.

THE question of the comparative healthiness of block-dwellings for the working classes is one that is exciting considerable interest at the present time in consequence of the number of these buildings which are being erected for the housing of persons turned out by various Metropolitan clearances. It has recently been stated that the mortality in the buildings of the Improved Industrial Dwellings Company for the year 1896, including deaths of tenants who died in hospitals, was only 9 9 per 1,000, and that in regard to the "Sandringham" Buildings, in Charing Cross Road, it was as low as 4·65, the average for nine years having been 9·25 per 1,000; while in the courts and alleys in the neighbourhood of Drury Lane the death-rate was as high as 34·67 per 1,000. These figures are very interesting, but they prove too much. It is impossible to admit for a moment that any health conditions could produce a death-rate of 4·65 per 1,000. It would, at that rate, take an average of 215 years to kill off the present inhabitants, and as we suppose that babies sometimes die even in block-dwellings, we may imagine to what an age some of these Methuselahs would have to live to make up this average span of life! It cannot be too strongly impressed upon people that such statistics have but the faintest connection with health conditions; what they show is that certain block-dwellings attract a peculiarly select set of tenants—that is all.

### Woman as a Milk Producer.

THE use of human milk in the rearing of human infants is much to be commended, and although, by dint of care and some intelligence, the artificial rearing of infants is often attended with great success, there can be no doubt that in the majority of cases the natural nutriment, if of good quality, is by far the best form of food in the early days of infancy. Considerable interest therefore attaches to the observations lately reported by Dr. Budin to the Obstetrical Society of France on "The Production of Milk by Wet-Nurses." In the ward for nurslings at the Maternity Dr. Budin had had as many as fifty children, in addition to the fourteen children belonging to the wet-nurses employed, and the problem he had to solve was how to make the milk of fourteen women do for sixty-four children—in other words, how to make the most out of these women as human "milkers." By a kind of systematic training he found that the milk was gradually increased, and he was able to satisfy himself that this increase in quantity was not brought about at the expense of quality. He found also that an indispensable condition for obtaining large quantities of milk was to require the nurses to suckle the infants freely. The more the milk was drawn the more was the quantity supplied, within reasonable limits. All this is in accordance with what is well known in regard to the yield of milk by cows. It is generally recognised in dairies that for the regular and abundant secretion of milk it is absolutely necessary that the supply should be thoroughly exhausted at each milking, and that if the milker is negligent, and does not take the trouble to empty the udder every time, the secretion rapidly ceases. Perhaps this is the explanation of the fact that to prevent the mother's milk from drying up it is often of great benefit to change the child, and to give a woman who has, perhaps, barely enough for one child one or two other babies to suckle. The result not infrequently is that a plentiful flow results, the reason being that the one child has not properly emptied the breasts, which have in consequence tended to dry up. To many women all this will sound very horrible—lowering to the sex, and all the rest of it. Women undoubtedly exist who even look upon maternity as a somewhat degrading affair, and what they will say to this use of women as milk producers we do not know. We do know, however, that woman's milk is the proper food for human infants, and we are inclined to think that a woman may be doing a nobler thing, and certainly be doing better for humanity, by so utilisng her milk-producing capacity as to set two or three children on their feet for life, than were she to employ her powers, say, in writing a novel.

### Police Cases at Hospitals.

A QUESTION of considerable interest both to hospitals and to the public has been raised in Dublin by the suicide of a man who had been refused admission to Mercer's Hospital on the ground that he was suffering from delirium tremens. It is stated that it is a rule of this institution not to admit patients affected with this disease without payment, unless in the case of the police, and many people are asking why any limitation should be put on the admission of urgent cases so long as there is room. Without referring any further to this individual case, we may express a doubt whether it is even a good plan to impose upon house surgeons any restrictions as to the admission of urgent cases, which, in fact, by their variety, defy all attempts at classification. In justification, however, of those institutions where such rules exist, it should be stated that in many cases they have been drawn up in the interests of the patients, and to protect the hospitals from certain very obvious abuses, chief among which is the tendency of the police to make use of hospitals in cases which would much more appropriately fall into the hands of their own surgeons. This is a practice which imposes upon charity in several directions. Hospital managers, responsible as they are for the expenditure of charitable funds, not unnaturally object to seeing them used for mere police purposes; then they properly enough dislike the sudden intrusion of riotous and noisy cases, by whom the patients are disturbed, especially when, as in the case of delirium tremens, the disease is self inflicted, and requires detention and control rather than active treatment. In still another way police cases very seriously disturb hospital arrangements, interfering with the treatment of the patients, and involving the institution in considerable expense. It is in regard to these cases chiefly that house surgeons are dragged off to attend inquests, police courts, sessions, and assizes, and hospital managers well know how completely this disorganises the working of any institution, besides the expense of providing substitutes. We cannot, then, wonder at the existence of restrictive rules at some hospitals, however much we may doubt their propriety.

